# Epigenetics and microRNAs in UGT1As

**DOI:** 10.1186/s40246-021-00331-6

**Published:** 2021-05-25

**Authors:** Cui-Lan Meng, Wei Zhao, Dan-Ni Zhong

**Affiliations:** Department of Pediatrics, The First Affiliated Hospital of Guangxi Medical University, No. 6 Shuangyong Road, Nanning City, Guangxi China

**Keywords:** UDP-glucuronosyltransferase (UGT)1As, Epigenetics, miRNA, Drug-metabolizing enzymes, Posttranscriptional regulation

## Abstract

UDP-glucuronosyltransferases (UGTs) are the main phase II drug-metabolizing enzymes mediating the most extensive glucuronidation-binding reaction in the human body. The UGT1A family is involved in more than half of glucuronidation reactions. However, significant differences exist in the distribution of UGT1As in vivo and the expression of UGT1As among individuals, and these differences are related to the occurrence of disease and differences in metabolism. In addition to genetic polymorphisms, there is now interest in the contribution of epigenetics and noncoding RNAs (especially miRNAs) to this differential change. Epigenetics regulates UGT1As pretranscriptionally through DNA methylation and histone modification, and miRNAs are considered the key mechanism of posttranscriptional regulation of UGT1As. Both epigenetic inheritance and miRNAs are involved in the differences in sex expression and in vivo distribution of UGT1As. Moreover, epigenetic changes early in life have been shown to affect gene expression throughout life. Here, we review and summarize the current regulatory role of epigenetics in the UGT1A family and discuss the relationship among epigenetics and UGT1A-related diseases and treatment, with references for future research.

## Introduction

UDP-glucuronosyltransferases (UGTs) are membrane proteins present in the endoplasmic reticulum. Human UGTs consist of a phase II metabolic enzyme family of 19 functional enzymes that compose four subfamilies: UGT1 (UGT1A), UGT2 (UGT2A and UGT2B), UGT3, and UGT8 [1, 2]. The UGT1A family is located on chromosomal band 2q37. The first exon encodes a specific sequence of the UGT1A functional protein, and the conserved 2–5 exons jointly encode the same sequence of UGT1A without specificity [3, 4]. The composition pattern of UGT2A1 and UGT2A2 was similar to that of UGT1A, while UGT2A3, UGT2B, UGT3, and UGT8 were encoded by a single gene with 6 independent exons [5, 6] (Table 1). Most endogenous and exogenous compounds (including bilirubin, steroid hormones, and commonly used drugs such as acetaminophen, analgesic morphine, and SN-38) need to be transformed and metabolized by UGT-mediated glucuronidation to play a role in or be successfully excreted from the body. Although UGT3 and UGT8 may provide relatively small contributions to drug metabolism [13], their other functions have been gradually explored [10, 11]. As one of the most abundant UGTs in the body, UGT1As are considered to participate in at least 50% of drug glucuronidation [14]. They can mediate the metabolic elimination of drugs for cancer [5], acquired immune deficiency syndrome (AIDS) [15, 16], organ immune rejection [17], glomerulonephritis (axitinib) [18], and leukemia (cytarabine) [19] treatments, among others. However, obvious tissue-specific and individual expression differences exist among the subtypes of UGT1As, leading to the occurrence of diseases (such as Crigler–Najjar syndrome types I and II (CN1 and CN2, respectively)) [20] and complexities in drug treatment dosages (such as therapeutic failure or drug toxicity) [21]. Although UGT1A gene polymorphisms, single-nucleotide polymorphisms (SNPs), and differences in transcriptional regulation may also lead to changes in transcription and/or enzyme activities in UGT1As, they cannot fully explain the tissue-specific expression of UGT1A and the asymmetry of mRNA and translation protein levels of UGT1As [22]. Human cell types contain the same genetic information of DNA sequences, but the expression of genes in cells or at different developmental stages is quite different. This tissue-specific or dynamic expression pattern indicates that in addition to the genome, other regulatory factors play important roles.

**Table 1:** UGT gene information table

Name	Distribution (kb)	Locus	Sugar donor	Isoform	Substrates	Function	References
UGT1	200	2q37	UDP-glucuronic acid	UGT1A1, 1A3-1A10	Endogenous lipophilic compounds: bilirubin, steroid hormones, bile acids, and fatty acids Exogenous lipophilic chemicals: carcinogens, environmental toxicants and pollutants	Detoxify and clear of endogenous and exogenous lipophilic compounds	[7, 8]
UGT2	1500	4q13	UDP-glucuronic acid	UGT2A: UGT2A1-2A3 UGT2B: UGT2B4, 7, 10, 11, 15, 17, 28	Same as above	Same as above	[3]
UGT3	115	5p13.2	UDP-N-acetylglucosamine, UDP-glucose, UDP-xylose	UGT3A1, UGT3A2	Ursodeoxycholic acid, 17 alpha-estradiol, 17 beta-estradiol, 4-nitrophenol, 5-1-naphthol	Responsible for the formation of N-acetylglucosaminides of UDCA and other compounds	[6, 9]
UGT8	Over>40kb	4q26	UDP-galactose	UGT8	Ceramide, deoxycholic acid, chenodeoxycholic acid, cholic acid, hyodeoxycholic acid, 4 ursodeoxycholic acid	1. Responsible for the biosynthesis of galactosylceramide and psychosine 2. A modulator of bile acid homeostasis and signaling	[10–12]

The multilevel regulation of UGTs can be summarized as pretranscription, transcription, posttranscription, and posttranslational regulation. Epigenetics is an important mechanism of the pretranscriptional regulation of UGTs, which leads to the activation or inactivation of gene functions through DNA methylation, histone modification, chromatin remodeling, and other regulatory mechanisms without changing the nucleotide genetic code sequence [23–25]. Transcriptional regulation of UGTs is mainly controlled by a combination of tissue-specific factors [26] (such as caudal-related homeodomain protein 2 (CDX2) and hepatocyte nuclear factors HNF1a) and ligand-activated transcription factors (TFs) with cis-regulatory elements (CREs) in gene promoters [7]. Posttranscriptional regulation of UGTs is mainly mediated by miRNAs, which regulate most mRNAs in the body. Under the action of the RNA-induced silencing complex (RISC), the miRNA seed sequence and target gene mRNA 3′-UTR sequence bind via complementary pairing to regulate gene expression through translation inhibition or mRNA degradation [27–29]. Posttranslational modification of UGTs mainly changes the structure and function of the protein through phosphorylation [30], glycosylation [31], protein-protein interaction [32, 33], etc., thus affecting the glucuronidation ability of UGTs.

In the multilevel regulation of UGTs, transcriptional and posttranslational regulation have been the best studied [34]. Because UGT1As are one of the most abundant UGTs in vivo, this review summarizes the pretranscriptional regulation (epigenetics) and posttranscriptional regulation (miRNA) of UGT1As. Then, we discuss how miRNAs, as epigenetic regulators, are in turn regulated by epigenetics. Most interestingly, researchers have found that epigenetic changes early in life can have a long-lasting effect on gene expression in adulthood and are an important part of living organisms adapting to the environment, producing immune memory, and responding to individual differences in internal and external environments [35, 36]. In general, epigenetics is a heritable change in gene expression and cell phenotype. In Cavalli and Heard’s view, epigenetics can include a process known to participate in epigenetic inheritance, even if it does not involve epigenetic memory itself [37].

## Epigenetics in UGT1AS

### DNA methylation profiles in UGT1As

**Fig. 1:**
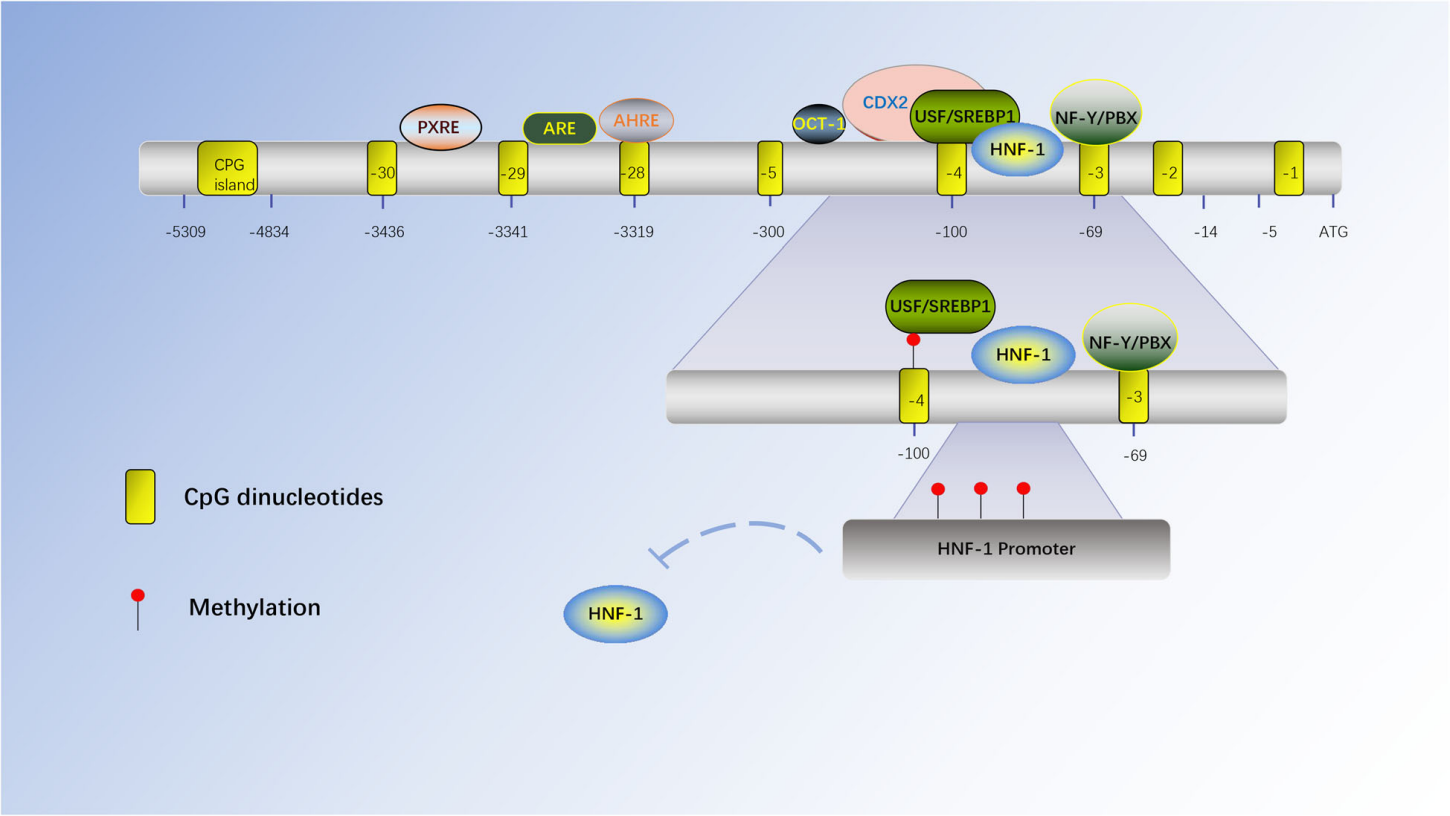
Schematic diagram of the regulation of UGT1A1 by DNA methylation

As the earliest recognized and most studied epigenetic mechanism, DNA methylation has attracted substantial attention partly because the advantage of maintaining unmethylated DNA in some organs is characteristic of organ-specific gene expression [38]. DNA methylation silences gene expression by recruiting transcription inhibitors (methyl-CpG binding proteins) to compete with transcription factors (TFs) for promoter binding sites or by directly inhibiting the binding of TFs to promoters [39, 40]. Numerous TFs regulate UGT1As. These TFs are widely expressed in various organs of the body, but even if TFs in these organs can upregulate the expression of the UGT1A gene, the expression of UGT1As is insufficient. UGT1A expression has obvious tissue specificity. For example, UGT1A9 is stably expressed in the liver and kidney, while UGT1A7, UGT1A8, and UGT1A10 are expressed only in the gastrointestinal tract [41]. The expression patterns of UGT1As give different organs a differential glucuronidation profile in internal and external substances. Among factors influencing unstable gene expression, epigenetic regulation of DNA methylation plays an important role (Fig. 1). The effects of DNA methylation on gene expression, drug activity, and metabolism are part of the cause of drug resistance in clinical therapy. Fully understanding the methylation of the UGT1A1 gene may also provide a regulatory mechanism for reversing drug resistance.

Studies have shown that the CpG island of the UGT1A1 promoter is hypermethylated in hepatogenic HuH-7 cells without UGT1A10 expression, while its gene promoter is hypomethylated in intestinal LS180 cells expressing UGT1A10 [42]. In HuH-7 cells, the TFs hepatocyte nuclear factor 1 alpha (HNF1α) and caudal type homeobox 2 (Cdx2) play a role in promoting UGT1A10 gene expression only in the presence of 5-aza-2′-deoxycytidine (5-Aza-dC), an inhibitor of DNA methylation [42]. Similarly, UGT1A1 is expressed in the liver but not in the kidney [43, 44]. Oda et al. showed in experiments that the UGT1A1 gene promoter CpG-rich region (−85 to +40) in the kidney is highly methylated compared with that in the liver, histone H3 is hypoacetylated, and the degree of HNF1α enrichment is low. This suggests that DNA methylation is an important reason for the difference in the distribution of UGT1A1 in vivo [43]. Although most studies report methylation of the UGT1A1 gene promoter, which may inhibit expression of UGT1A1 in tissues, Yasar et al. obtained different results in a group of healthy human liver specimens. They found that UGT1A1 5′-flanking-region DNA methylation has obvious individual differences [45]. Among them, higher methylation of the 4th position upstream of the UGT1A1 ATG initiation codon (-4CpG) located in the upstream stimulation factor (USF) of the response element was associated with higher UGT1A1 expression and glucuronidation activity in human liver samples. Thus, the DNA methylation site could be the reason for the difference in the UGT1A1 expression level, and it deserves our attention.

#### DNA methylation regulation at UGT1As and links to disease

UGT1As are important functional enzymes that mediate glucuronidation, which is necessary for metabolizing and transforming many drugs. UGT1A1 is the most abundant enzyme in the liver, and it is the only functional enzyme with bilirubin glucuronidation activity [46]. UGT1A1 is also an efficacious and resistance-related enzyme of irinotecan in the treatment of colorectal cancer. Currently, DNA methylation can inhibit the expression of most drug-metabolizing enzymes [47].

Among 50 patients with primary colon tumors, Gagnon et al. found that most showed low UGT1A1 expression and that the gene promoter methylation level correlated negatively with UGT1A1 expression [48]. In addition, hypermethylated colon cancer HCT116 cells were highly sensitive to 7-ethyl-10-hydroxycamptothecin (SN-38, the active metabolite of irinotecan), while hypomethylated HT-29 cells were not. Subsequently, a study by Belanger et al. also showed that DNA methylation of the UGT1A1 gene promoter inhibited the expression of UGT1A1 in colon cancer cells [49]. In addition, the higher the glucuronidation activity in colon cancer cells with low UGT1A1 methylation was, the higher was the inactivation of SN-38. This effect is related to drug resistance in the treatment of colon cancer. In addition, they found that in HCT116 cells lacking HNF1α, the expression of this factor could be detected by adding a DNA methyl inhibitor. DNA methylation may indirectly downregulate the activation of target genes by inhibiting the expression of TFs. In 2014, Xie et al. also reported that in 99 patients with colorectal cancer, UGT1A1 promoter methylation could inhibit gene expression, thereby affecting the metabolic level of irinotecan (CPT-11) [50]. In summary, DNA methylation is expected to be a new target for drug resistance mechanisms.

Notably, the degree of methylation of the -4CpG site found by Yasar et al. correlates positively with UGT1A1 expression, which is contrary to results from other studies [45]. They believe that this is related to the detection method, as bisulfite sequencing is defective and will confuse 5mC and other modified cytosines [51]. Furthermore, the stress mechanism caused by environmental changes in downregulated UGT1A1 expression in different cells is also different. As previously reported, downregulation of UGT1A1 in the human liver leads to an increase in the level of bilirubin, which in turn acts as a ligand of AhR to promote the expression of UGT1A1 [52]. Moreover, Oda et al. believes that nonparenchymal cells in intact liver samples do not express UGT1A1, which has an impact on the experimental results [42]. Furthermore, an unknown regulatory mechanism in the tumor microenvironment that cuts off the relationship between methylation and UGT1A1 expression cannot be ruled out.

DNA methylation of gene promoters does not always regulate UGT1As. In the experiment of Oda et al., UGT1A9 was expressed only in human hepatocytes, but the DNA methylation status was not significantly different in the liver and small intestine [42]. Moreover, the research results of Yasar et al. and Oda et al. also differed with regard to whether DNA methylation affects the binding of HNF1α nearby [42]. However, this has laid the foundation for us to think about and explore the relationship between DNA methylation and its nearby gene activation cofactors. Therefore, in the UGT1A regulatory network, exploring whether the regulatory role of DNA methylation is antagonistic or agonistic is necessary.

### Histone modifications of UGT1As

Histone modifications are also an important means of epigenetic regulation of gene expression. Histone modifications include acetylation, methylation, phosphorylation, and ubiquitination [53–55]. These modifications are mainly regulated by histone-modifying enzymes, which cause changes in the chromatin structure and affect the binding of histones to DNA [56]. Histone modification is commonly coordinated with DNA methylation to regulate gene expression. For example, histone modification and DNA methylation cooperatively regulate the expression of UGT1A1 in the kidney and inhibit the expression of cytochrome P450 family 24 subfamily A member 1 (CYP24A1) in prostate cancer cells [57]. Of course, histone modifications can also work independently. Due to the poor development of the UGT1A1 enzymes that metabolize bilirubin in early life, newborns are prone to hyperbilirubinemia, and serious cases of hyperbilirubinemia can be life-threatening. Nie et al. showed that histone modification can regulate the expression of UGT1A1 in the liver during development. They found that high expression of UGT1A1 in the adult liver was related to enrichment of the transcriptional activation marker H3K4me2. The inhibition of fetal UGT1A1 expression was consistent with the high enrichment of enhancer of zest homolog 2 (EZH2) of the UGT1A1 promoter and the transcriptional suppression marker H3K4me3 (catalyzed by EZH2) [58]. Children with hyperbilirubinemia are more likely to develop infection during clinical treatment. Infection and hyperbilirubinemia appear to promote each other through histone modifications. The inflammatory factors IL-1 and lipopolysaccharide (LPS) can cause deacetylation of histone H4 in the promoter of the constitutive androstane receptor (CAR) gene, which is the upstream driver of UGT1A1, thus downregulating the expression of UGT1A1 and further aggravating the accumulation of bilirubin [59]. Phenobarbital (PB), as a typical CAR agonist, has been shown to increase the levels of UGT1A6 and UGT1A7 in the brain by activating oxidative stress, increasing H3K4me3 and reducing H3K9me3 (related to gene silencing) rather than inducing CAR [60].

Overall, the occurrence of disease and drug metabolism has been shown to have significant sex differences [61]. Studies have found that women are more susceptible than men to some liver diseases, such as autoimmune hepatitis and primary biliary cholangitis, while men account for a higher proportion of cirrhosis, chronic viral hepatitis, and liver cancer than women [62, 63]. Because UGT-mediated glucuronidation generally occurs more often in men than in women, the clearance rate of acetaminophen in men is higher [64]. The mechanisms of these phenomena may be related to the differences in the regulation of gene expression in the liver, especially those (such as UGTs) regulated by sex hormones [65]. Kalthoff et al. investigated the relationship between estrogen receptor alpha (ERα) and the sex-specific expression of UGT1As and found that chromatin remodeling caused by histone modification was involved in the sex-differential expression of UGT1As [66]. ERα binds to the xenobiotic response element (XRE) of UGT1As and inhibits transcription of the UGT1A gene by recruiting histone deacetylases 1 and 2 (HDAC1 and HDAC2, respectively). In the study by Tan et al., histone lysine crotonylation (Kcr) marks active promoters and may control the differentiation of male germ cells [67]. These findings indicate that histone modifications play an increasingly important role in the differential expression of genes.

Organismal development is a complex process. Factors that regulate and are regulated often coexist and complement each other. Histone modifications are regulated while regulating gene expression. Knockout of the TF HNF1α has been confirmed to reduce the enrichment of H3K4me2 in the promoter region of UGT1A1 [58]. Oxidative stress can regulate histone-related enzymes to upregulate the level of H3K4me2 [68]. Notably, epigenetic drugs used in clinical treatment, such as the histone deacetylase inhibitors suberoylanilide hydroxamic acid (SAHA) and belinostat, are also mainly metabolized by the UGT1A family, and the expression level of UGT1As can affect their therapeutic effect [69, 70].

## miRNA in UGT1As

### miRNA profiles in UGT1As

MicroRNAs (miRNAs) are single-stranded RNA molecules consisting of 19–25 nucleotides. Studies have shown that miRNAs can regulate the expression of drug-metabolizing enzymes, including UGTs [71, 72]. The gene polymorphism of UGT1As can affect glucuronic acid acidification. In SNPs of UGT1As, rs10929303, rs1042640, and rs8330 were found to be related to the activation of glucuronic acid, among which rs8330 has a higher level of glucuronidation [73]. The frequency of rs8330 is higher in Africans and lower in East Asians than in Europeans. However, no difference was found in glucuronidation between Venda (South African) and a white population [73]. Another example is that the glucuronidation of Chinese people living in Hong Kong is lower than that of European whites, but no difference in glucuronidation was found between Chinese people living in Australia or Canada and European whites [74–76]. Interestingly, these common SNPs occur on UGT1A 3′-UTRs, which have been proven to hinder or promote miRNA binding [77]. Thus, we have a good reason to suspect that posttranscriptional regulation of miRNAs plays a role in this difference.

Obviously, increasing attention is being focused on miRNA research, but few studies exist on the regulation of the gene expression of UGT1As. In recent years, miRNA pharmacogenomics has been proven to affect pharmacokinetics and toxicity and reduce the therapeutic response of drugs, playing an important role in individualized clinical medication and the prevention of adverse consequences [78, 79]. Therefore, we must summarize and consider the relationship between the two. At present, cells commonly used for the study of miRNA and UGT1As are human primary hepatocytes, hepatoma cell lines (HepG2 and HuH-7, which express high levels of UGT1As and are easy to transfect), a human embryonic kidney cell line (HEK293, which is easy to transfect, stably expresses several UGT1As, and is a commonly used cell line for studying foreign genes), and colon cancer cell lines (LS180, LS174T, and CaCo-2, which have high UGT1A expression and glucuronidation activity). At the same time, TFs such as aryl hydrocarbon receptor (AhR) that regulate UGT1As can be easily detected.

With research on the relationship between miRNAs and UGT1As, many miRNAs have been proven to affect the expression of UGT1As (Table 2). Papageorgiou et al. used the luciferase reporter gene method to screen out miRNAs that could reduce the fluorescence activity of the UGT1A 3′-UTR by more than 30% and verified the respective miRNA response elements (MREs) [77]. Among them, miR-141-3p can downregulate the mRNA and activity expression of UGT1A1 and UGT1A6 in LS180 and human hepatocytes. Studies have found that miR-141-3p can also downregulate the mRNA and activity expression of UGT1A1 and 6 in HEK-293 cells, HuH-7 cells, and CaCo-2 cells [80]. However, in liver tissues that have been genotyped, overexpression or inhibition of miR-141-3p had no effect on UGT1A1 and UGT1A6. Dluzen et al. also found that miR-491-3p can downregulate the expression of UGT1As in HuH-7 cells (UGT1A1, 3, 6) and human hepatocytes (UGT1A3, 6) but has no effect on the expression and activity of UGT1A1 in HepG2 [81]. This suggests that under different circumstances, the same miRNA has different effects on the same UGT1A subtype. Wang et al. found that miR-298 and miR-491-3p could regulate the expression of UGT1As in the Han Chinese population [82]. MiR-298 can regulate UGT1A3 and UGT1A4 in normal human liver tissues but has no effect on UGT1A1, UGT1A6, and UGT1A9. MiR-491-3p negatively regulates UGT1A3 and UGT1A4 in Han Chinese hepatocytes but has no correlation with UGT1A1, UGT1A6, and UGT1A9. This suggests that a miRNA can only regulate partial UGT1A subtypes in the same cells. In studying the effect of miRNA on UGT1As, Papageorgiou et al. found the effect of SNPs on miRNA binding to UGT1As. The rs8330 SNP of the UGT1A 3̸′-UTR can alter miR-1286 MRE and affect the binding of miRNA, and rs10929303 can form the seed sequence of miR-21-3p and enhance its binding to the target gene mRNA [77]. More regulation rules between miRNAs and UGT1As need to be further studied.

**Table 2:** UGT1As regulated by miRNAs

miRNAs	Target cells	Target UGT1As	References
miR-21-3p, miR-103p, miR-141-3p, miR-200a-3p, miR-376b-3p	LS180	UGT1A1, UGT1A6	[77]
miR-21-3p, miR-141-3p, miR-200a-3p	Human hepatocytes	UGT1A1, 4, 6, 9	[77]
miR-141-3p	HEK-293	UGT1A1, 3, 4, 6, 7, 9	[80]
miR-141-3p	HuH-7	UGT1A1, 4, 6, 8, 10	[80]
miR-141-3p	CaCo2	UGT1A1, 4, 6, 9	[80]
miR-491-3p	HuH-7	UGT1A1, 3, 6	[81]
miR-491-3p	Human hepatocytes (western counties)	UGT1A3, UGT1A6	[81]
miR-491-3p	Human hepatocytes (Chinese Han population)	UGT1A3, UGT1A4	[82]
miR-298	LS174T	UGT1A1, 3, 4, 9	[82]
miR-298	Human liver tissues	UGT1A3, UGT1A4	[82]
miR-548d-5p	Human hepatocytes	UGT1A1	[83]
miR-548d-5p	HepG	UGT1A1	[83]
miR-200a-3p, miR-183-5p	HCC	UGT1A9	[84]
miR-375	LS180	UGT1A1, UGT1A6	[85]
miR-375	Human hepatocytes	UGT1A	[85]

### miRNA regulation of UGT1As and links to disease

In recent years, miRNAs have been used to predict disease because they are often differentially expressed in healthy individuals versus those with a disease or disorder and have therefore become the focus of biomarker research [86]. Douglas et al. [83] studied the role of the regulatory relationship between miR-548d-5p and UGT1A1 in the treatment of female complex regional pain syndrome (CRPS) with ketamine administration. Patients with a low efficacy of ketamine treatment exhibited downregulated miR-548d-5p expression before drug treatment. UGT1A1 and glucuronidation activities were significantly reduced in HepG cells transfected with miR-548d-5p. Previous studies have shown that cytochrome P450 family 3 subfamily A member 4 (CYP3A4) and UGT regulated by miR-548d-5p are two key enzymes in the biotransformation of ketamine [87, 88]. In the treatment of CRPS with ketamine, miR-548d-5p mainly binds to the 3′-UTR of UGT1A1 but not to the 3′-UTR of CYP3A4. The former binding inhibits the expression of UGT1A1 mRNA and protein in hepatocytes, while glucuronidation mediated by UGT1A1 plays a crucial role in the metabolism and duration of efficacy with ketamine in vivo. In patients with a low efficacy of ketamine treatment, low expression of miR-548d-5p will lead to a high activity of UGT1A1 and a high level of glucuronidation in hepatocytes, which will accelerate the metabolic clearance of ketamine drugs, thus reducing the therapeutic effect [83]. miRNAs that can play a predictive role in therapeutic drugs related to UGT1A metabolism also include miR-200a-3p and miR-183-5p. Ge et al. [84] found that among patients with hepatocellular carcinoma (HCC) who were treated with sorafenib after surgery, patients with a good prognosis had high expression of UGT1A9. Subsequently, a fluorescence reporter gene confirmed that miR-200a/-183 had a negative posttranscriptional regulatory effect on UGT1A9 expression. A high level of miR-200a/-183 indicates a low level of UGT1A9, thus reducing the formation of sorafenib β-D-glucuronic acid in HCC and reducing the efficacy of drugs.

Taken together, these results suggest that miR-548d-5p and miR-200a/-183 regulate the metabolism of drugs in vivo by downregulating the expression of UGT1A1 and UGT1A9, respectively, thereby affecting the therapeutic effect. This suggests that miR-548d-5p and miR-200a/-183 can be used as biomarkers to predict the therapeutic effect of drugs. In addition, miR-548d-5p and miR-200a/-183 regulate the metabolic degree of the drug in the body by downregulating UGT1As.

miRNAs (such as miR-375) can also regulate UGT1As through indirect mechanisms (TFs) to participate in drug metabolism. Individual differences exist in the therapeutic dose of acute liver failure (ALF) induced by acetaminophen. Because the key enzyme for acetaminophen metabolism and clearance is UGT1A, the variation in acetaminophen glucuronidation between individuals causes poisoning susceptibility in some people [89, 90]. Through a transcriptome association analysis, Papageorgiou et al. found that the expression level of miR-375 in the group with low UGT1A activity was significantly higher than that in the group with high UGT1A activity [85]. At the same time, the binding site of miR-375 that is related to UGT1A activity and that is differentially expressed was found to be not on the UGT1A 3′-UTR but on the mRNA of AhR, which is a TF that has been clearly shown to regulate UGT1As [85]. In addition, overexpression of miR-137 in LS180 cells reduced the expression of the AhR target genes UGT1A1, UGT1A6, and cytochrome P450 family 1 subfamily A member 2 (CYP1A2). Thus, miR-137 indirectly downregulates the activity of UGT1As by inhibiting AhR expression, resulting in low acetaminophen glucuronidation activity. Therefore, miR-137 can increase ALF triggered by acetaminophen. Court analyzed the phenotype of acetaminophen glucuronidation activity in the human liver bank (Local Disease Research Center or Liver Tissue Procurement and Distribution Service Center [64]) and genotyped the metabolic enzymes UGT1A1, UGT1A6, and UGT1A9. They found that SNP rs8330 located on the 3′-UTR shared by human UGT1A is a protective gene for paracetamol-induced liver injury, and it can increase the glucuronidation activity of acetaminophen and reduce the risk of ALF [73]. Moreover, as mentioned in that study, the rs8330 and MRE SNPs in miR-1286 overlap and influence each other [77]. Therefore, does the lack of regulation of miR-1286 caused by rs8330 play a role in the risk of ALF? This situation is worthy of our attention. These results suggest that miR-375, miR-1286, and UGT1A genotyping play an important role in formulating individualized medications and preventing adverse reactions to drugs.

Among the diseases caused by dysregulation of UGT1A1 activity, treatment of CN1, which is an inherited metabolic disease (IMD) caused by the complete loss of UGT1A1 activity, has always been a puzzling problem for health care providers. In the treatment of CN1, liver transplantation is the only way to permanently restore the expression and activity of UGT1A1. Previously, gene therapy in Gunn rats (a model of CN-I disease) often resulted in treatment failure, which may be due to the immune rejection of cytotoxic T cells caused by viral vectors triggering antigen-presenting cells (APCs) [91]. MiR-142, which is specifically expressed in hematopoietic cells and APCs, can make lentiviral gene vectors evade the immune response. miR-142 can permanently cure hemophilic mice in gene therapy [92]. The ability of miR-142 to degrade APC mRNA plays a vital role in gene therapy. Schmitt et al. have shown that the addition of miR-142 to lentiviral vectors carrying UGT1A1 cDNA can avoid the clearance of target protein cells by immune rejection [93]. In addition, miR-142 can stabilize the immune tolerance of gene therapy mice by recruiting and upregulating the expression of regulatory T cells (Tregs) [94]. Therefore, in lentiviral gene therapy CN1 mice, miR-142 can combine with the APC mRNA 3′-UTR to degrade APCs and upregulate Tregs to resist immune rejection in the body to achieve stable and long-term expression of the UGT1A1 gene for treatment. These studies provide a new approach for gene therapy engineering.

miRNAs are important for gene regulation, but their role in UGT1A-mediated drug metabolism research has just begun. Functional genomics and transcriptomics studies have demonstrated the direct or indirect regulation of UGT1As by miRNA. Nevertheless, the specific functions of many miRNAs related to UGT1As are still unclear and need to be investigated. For example, in the research of Papageorgiou et al., miR-141-3p can inhibit the mRNA level and activity of UGT1A1 in the human liver [77]. However, Tatsumi et al. found no significant correlations between miR-141-3p and UGT1A1 in genotyped human liver tissues [80]. The reasons for this discrepancy need to be explored and suggest the complexity of gene regulation. As found by Takagi et al., miR-24 regulates hepatocyte nuclear factor 4 alpha (HNF4α) not by binding to the noncoding 3′-UTR of the target gene HNF4α mRNA but by binding to the mRNA coding region [95]. In addition, among the numerous miRNAs that can regulate UGT1As, many miRNAs are known to indirectly regulate UGT1A expression by regulating TFs [96, 97]. When studying the regulation of UGT1As by miRNAs in cell lines, whether the cell line used has polyploidy, chromosomal breaks and rearrangements, or duplications and deletions, which will cause differences in miRNA regulation, cannot be ignored. However, the fact that miRNAs have achieved initial results in gene therapy cannot be denied.

### UGT1As-related miRNAs are epigenetically regulated

Wang et al. found that approximately 50% of miRNA genes in vivo are associated with CpG islands, indicating that the expression of miRNA is affected by DNA methylation [98]. In other diseases, the expression of some miRNAs related to UGT1A regulation has been shown to be influenced by DNA methylation. In Hirschsprung’s disease (HSCR), CpG island methylation in the promoter region of the miR-141 gene leads to its downregulated expression [99]. In human temporal lobe epilepsy, miR-375 was found to be highly sensitive to DNA methylation [100]. MiR-375 expression has also been found to be regulated by DNA methylation in lung cancer, metastatic melanoma, and type 2 diabetes mellitus [101–103]. A hypermethylation status of the miR-200 family gene promoter was also found in invasive bladder cancer [104]. However, with regard to the expression of UGT1As, the epigenetic status of these miRNA gene promoters remains to be studied.

## Epigenetics and genetic “memory”

In recent years, the phenomenon of epigenetics causing genes to produce long-term “memory” has become increasingly familiar. For example, in cases related to drug-metabolizing enzymes, the treatment of newborn mice with CAR-activating drugs can lead to a persistent increase in H3K4me2 and H3K4me3 in the cytochrome P450 family 2 subfamily B member 10 (CYP2B10) gene. Studies have shown that this will reduce the sensitivity of adult mice to related drugs [105]. Phenobarbital therapy at the beginning of life to produce histone modification is a key factor in the continuous expression of the P540 gene and the induction of enzyme activity in adult mice [106]. After birth, the methylation status of fibroblast growth factor-21 (fibroblast growth factor-21 gene) can persist into adulthood and affect the obesity rate in adulthood [107]. This “memory” may indicate the persistence of epigenetic marks during cell mitosis. These examples provide hints as to the type of sustained effects of early epigenetic changes in UGT1As. Are these effects the cause of differences in individual pharmacokinetics in adulthood? To understand whether epigenetics truly has a “genetic” effect on gene expression, Cavalli and Heard suggest that we combine lineage tracing with single-cell “-omics” technologies to understand the gene expression history of the cell lineage [37, 108]. Thus, the permanent influence of early epigenetic changes is a double-edged sword in biological activities and is worth exploring.

## Conclusions and discussion

We have long known that the expression of the UGT1A family plays an irreplaceable role in mediating substance metabolism. Obviously, the research described above fully illustrates the important role of DNA methylation, histone modification, and miRNAs in UGT1A expression and drug metabolism in related diseases at the level of epigenetic regulation. Based on the potential reversibility of epigenetics, genomic therapy for UGT1A-related diseases also has a strong theoretical basis. At the same time, it reminds us not to ignore the study of epigenetic changes when studying the pharmacological effects of drugs, and these potential changes also have important roles. However, due to the difficulty of related research, the amount of literature available for analysis is limited, the conclusions of studies vary to some extent, and few relevant epigenetic drugs formally enter clinical treatment. Moreover, there are no reports about changes in UGT1A expression caused by early epigenetic changes. Further research should include multicenter specimens and a large patient group while using new technologies (lineage tracing with single-cell “-omics” technologies) to study the epigenetic “memory” of UGT1As. It is undeniable that with the development of more in-depth research, the regulatory network of epigenetics on UGT1As will become increasingly clear, thus providing a new mechanism for personalized medicine in clinical practice and increasing the understanding of the variability of the drug response.

The figure shows the CPG site of UGT1A1 and the location of the response elements of different transcription factors that have been verified in the laboratory [45, 49]. Some CPG sites are located just inside the transcription factor response element. Thus, CPG methylation can hinder the binding of TFs and affect the level of transcription. At the same time, TF gene promoter DNA methylation can inhibit the transcription of TFs itself, thereby affecting the transcription of downstream target genes.

## Data Availability

The datasets generated and/or analyzed during the current study are available in the (NAME) repository (PERSISTENT WEB LINK TO DATASETS).
